# Quantitative Trait Loci Mapping for Earliness, Fruit, and Seed Related Traits Using High Density Genotyping-by-Sequencing-Based Genetic Map in Bitter Gourd (*Momordica charantia* L.)

**DOI:** 10.3389/fpls.2021.799932

**Published:** 2022-02-08

**Authors:** Gurpreet Kaur, Mamta Pathak, Deepak Singla, Gautam Chhabra, Parveen Chhuneja, Navraj Kaur Sarao

**Affiliations:** ^1^School of Agricultural Biotechnology, Punjab Agricultural University, Ludhiana, India; ^2^Department of Vegetable Science, Punjab Agricultural University, Ludhiana, India

**Keywords:** bitter gourd, genotyping by sequencing, horticultural traits, quantitative trait loci, mapping

## Abstract

Bitter gourd (*Momordica charantia* L.) is an important vegetable crop having numerous medicinal properties. Earliness and yield related traits are main aims of bitter gourd breeding program. High resolution quantitative trait loci (QTLs) mapping can help in understanding the molecular basis of phenotypic variation of these traits and thus facilitate marker-assisted breeding. The aim of present study was to identify genetic loci controlling earliness, fruit, and seed related traits. To achieve this, genotyping-by-sequencing (GBS) approach was used to genotype 101 individuals of F_4_ population derived from a cross between an elite cultivar Punjab-14 and PAUBG-6. This population was phenotyped under net-house conditions for three years 2018, 2019, and 2021. The linkage map consisting of 15 linkage groups comprising 3,144 single nucleotide polymorphism (SNP) markers was used to detect the QTLs for nine traits. A total of 50 QTLs for these traits were detected which were distributed on 11 chromosomes. The QTLs explained 5.09–29.82% of the phenotypic variance. The highest logarithm of the odds (LOD) score for a single QTL was 8.68 and the lowest was 2.50. For the earliness related traits, a total of 22 QTLs were detected. For the fruit related traits, a total of 16 QTLs and for seed related traits, a total of 12 QTLs were detected. Out of 50 QTLs, 20 QTLs were considered as frequent QTLs (FQ-QTLs). The information generated in this study is very useful in the future for fine-mapping and marker-assisted selection for these traits in bitter gourd improvement program.

## Introduction

Bitter gourd (*Momordica charantia* L.) is one of the important vegetable crops, known as bitter cucumber, *karela*, bitter melon, and African pear (Basch et al., 2003). This vegetable crop belongs to family *Cucurbitaceae* and genus *Momordica* having chromosome number 2x = 2n = 22. Fruits and seeds of bitter gourd have some useful therapeutic properties and hence used for the treatment of various illnesses, such as stomach pain, fever, malaria, anemia, and coughs (Girón et al., 1991). It is a rich source of vitamins, carbohydrates, minerals, and possess anti-diabetic (Raman and Lau, 1996; Bakare et al., 2010), antioxidant (Krishnaiah et al., 2011), and anti-hepatotoxic (Semiz and Sen, 2007) properties. Bitter gourd is helpful in the antiviral HIV infection therapy (Lee-Huang et al., 1995) and it reduces the proliferation of cancer cells (Haque et al., 2011; Brennan et al., 2012).

The demand of the bitter gourd crop has increased over the years, so high crop yield is the pursuing direction of research for the vegetable breeders. Several workers reported significant variation for earliness related traits in bitter gourd (Dey et al., 2009; Khan et al., 2015). Apart from earliness, yield is another very important trait in bitter gourd which is complex in nature and is influenced greatly by flowering and fruit traits, such as number of staminate and pistillate flowers, number of fruits plant, length, diameter, and weight of fruits, plant vigor, and length of the vine of a particular genotype (Iqbal et al., 2016; Singh et al., 2017). A few quantitative trait loci (QTLs) have been reported in bitter gourd relevant to yield component traits (fruit weight, length, and diameter) and earliness (Kole et al., 2012; Wang and Xiang, 2013; Cui et al., 2018; Rao et al., 2018, 2021).

Marker-assisted breeding for agronomic traits is applicable because of genetic mapping as plants with desirable gene combinations can be selected at early generations. In genetic mapping studies of bitter gourd, major emphasis was on flower or fruit characteristics due to their agronomical importance. Some linkage maps were developed in bitter gourd with the aid of molecular markers which were then used for mapping for various traits. Kole et al. (2012) developed the first linkage map in bitter gourd using 10 AFLP markers. Five simply inherited loci for traits, such as fruit luster, fruit color, fruit surface structure, seed coat color, and stigma color were successfully detected. Five quantitative traits for the fruit length, number, weight, and width contributing to fruit yield were mapped. Subsequently, large number of QTLs for bitter gourd complex traits were detected and mapped on the linkage maps. Forty-three QTLs for 13 horticultural traits, first female flower node, female flower ratio, fruit thickness, fruit length, fruit diameter, fruit shape, fruit length pedicel ratios, fruit pedicel length, fruit numbers per plant, yield per plant, fruit weight, stem diameter, and length of internodes were investigated by using a linkage map developed from simple sequence repeats (SSR), AFLP, and sequence-related amplified polymorphism (SRAP) markers (Wang and Xiang, 2013). However, low marker density on these map limits the QTL mapping accuracy, which leads to low QTL mapping resolution (Yu et al., 2011).

Due to advancement in the next-generation sequencing (NGS) technologies and the declining cost of genotyping, it has become possible to develop high density SNP-based markers for the genotyping of mapping populations. Genotyping-by-sequencing (GBS) (Elshire et al., 2011) has been used widely for the development of linkage maps based on high density SNP markers and these maps have been utilized for the mapping of various useful traits in various species (Poland J.A. et al., 2012; Sonah et al., 2013; Spindel et al., 2013; Chen et al., 2014; Zhou et al., 2016). It increases the precision and potential of QTL mapping thus affecting both the detection and the resolution of QTLs. The GBS approach has been applied in crops having well sequenced reference genomes (Poland J. et al., 2012; Mascher et al., 2013) but GBS approach has also been successfully adapted for the mapping in crops without reference genomes sequences using *de novo* approach (Lu et al., 2013). In bitter gourd, different researchers used different mapping populations derived from contrasting parents for the development of linkage maps in independent studies (Urasaki et al., 2017; Cui et al., 2018; Rao et al., 2018). In these studies, a large number of SNP markers were generated and used for the mapping of QTLs for the horticultural traits.

In this study, we performed QTL analysis of nine traits evaluated over three growing seasons using a high-density genetic map containing of 3,144 SNP loci covering all the bitter gourd 11 chromosomes for the identification of QTLs to be utilized in bitter gourd breeding programs.

## Materials and Methods

### Plant Material (Mapping Population)

The experimental material for the present study consisted of female parent Punjab-14 and the male parent PAUBG-6. Punjab-14 is a cultivated variety released in 1985 by Punjab Agricultural University (PAU), Ludhiana at the national level. It possesses several salient features, such as small vines and small light green fruits having glossy appearance. These fruit traits of Punjab-14 are contrasting with the PAUBG-6 as it has long vines with dark green and fruits with matt appearance and pubescent stem. A total of 101 lines representing three generations of F_2_-derived lines (F_2:3_, F_2:4_, and F_2:5_) were developed by single-seed descent (SSD) method by Kaur et al. (2021). The field trials were conducted in the experimental farms of PAU Ludhiana (30^°^54′ N latitude, 75^°^48′ E longitude, and 247 m above sea level) for three growing seasons with two replications (six plants from each line) each in 2018 (Y1), 2019 (Y2), and 2021 (Y3) in randomized block design under insect proof net houses. The seeds of the population and their parents were sown in plug trays with cocopeat medium. About 25–30 days old seedlings were used for transplanting under insect-proof net houses as the parent Punjab-14 is susceptible to yellow vein mosaic disease. The seedlings were planted on well prepared hills 1.5 m wide beds with plant-to-plant distance of 45 cm. The observations on individual plants were recorded. All the recommended fertilizer doses, cultural practices, and plant protection measures were carried out in experimental plots to raise a successful crop (Anonymous, 2018).

### Phenotyping of Horticultural Traits

The bitter gourd horticultural traits investigated included earliness related traits, such as days to appearance of first female flower (DFF), days to appearance of first male flower (DMF), node to appearance of first female flower (NFFF), and days to fruit maturity (DFM), fruit related traits, such as fruit length (FL), fruit diameter (FD), and fruit weight (FW), and seed related traits, such as seed hardness (SH) and seed number (SN). These were recorded for parental lines and F_4_ (2018) and F_5_ populations (2019 and 2021) following DUS guidelines (Protection of Plant Varieties and Farmers’ Right Authority, India). DFF and DMF were counted from the date of transplanting till the appearance of the first flower. DFM was counted from the date of pollination to the date of harvesting of mature fruits while for NFFF, the nodes were counted on which the first female flower appeared. Fruit traits, such as FL, FD, and FW were recorded at the marketable stage (15 days after pollination). To observe the length and diameter of fruits, a vernier caliper was used to take measurements. For seed traits, SH and SN, data were recorded after harvesting. The seed hardness was measured in kilogram-force (kgf) using a Tablet Hardness Tester equipment and break point of seed coat was recorded. Briefly, 10 seeds were randomly selected from each plant. Moisture content was adjusted to give equal levels in all the seeds by keeping the samples in an oven at 39°C for 3--4 days. The data for earliness traits, such as DFF, DMF, and NFFF were recorded from six plants from each F_4_ line with two replications while the data for fruit traits, such as DFM, FL, FD, and FW were recorded by taking an average of five fruits per plant. Pearson’s correlations coefficients (*r*) between different traits under three different environments were calculated and plotted in R version 3.22.^1^ The standard deviation (SD), skewness, kurtosis, and coefficient of variation (CV) were calculated using Excel 2010. The ANOVA for randomized block design was carried out by using the following model.


Y=ijkm+g+ijb+keijk

Where,

Y_*ijk*_ = phenotypic value of the ijth genotype grown in the kth replication

m = general population mean

g_*ij*_ = effect of the ijth genotype, where I, j, = 1…g

b_*k*_ = effect of the kth replication, where k = 1…r

e_*ijk*_ = environmental effect.

Variance components were obtained by equating the mean squares as given below:


σe=2M3

σgy=2M-2M/3Y

σg=2M-1M/2ry

σp=2σg+2σe2

where σe^2^, σgy^2^, σg^2^, and σp^2^ are components of variance due to error (GE), genotype x environment interaction (GEIV), genotypes (GV), and phenotypes (PV), respectively. M_1_, M_2_, and M_3_ are the observed values of mean squares for the genotypes, interaction, and error, respectively (Fehr, 1987). Heritability, estimates of genotype × environment interaction (GEIV), genotypic coefficient of variation (GCV), and phenotypic coefficient of variation (PCV) were calculated using the following formulae (Singh and Choudhary, 1985). h^2^ = σg^2^/(σg^2^ + σe^2^)


GCV=√σg/2U¯

PCV=√σp/2U¯

Where $\bar{\text{U}}$ is the mean of the samples.

### Quantitative Trait Loci Mapping

The 101 F_4_ lines developed from the cross between Punjab-14 and PAUBG-6 were genotyped earlier by a GBS approach (Kaur et al., 2021) to map the yellow mosaic virus resistance in bitter gourd. The linkage map was constructed using the reference genome of genotype sequence OHB3-1 of bitter gourd (DNA Data Bank of Japan, accession nos. BLBB01000001–BLBB01000011) (Matsumura et al., 2020). As the parents have contrasting horticultural traits so this population was maintained at Punjab Agricultural University for the further evaluation of the useful horticultural traits in well maintained virus free net-houses. SNP based linkage map consisting of 3,144 SNP markers as reported by Kaur et al. (2021) was used for QTL mapping for earliness and fruit related traits.

To search for QTL for each trait, composite-interval mapping (CIM) was used in Windows QTL Cartographer 2.5 software (Wang et al., 2012). “Model 6 standard analyses” parameter setup was used with a walk speed of 0.5 cM. A blocked window size of 10 cm was chosen to exclude the closely linked control markers at the testing site along with “forward and backward” regression for the selection of the markers to control for the genetic background, up to five control markers. Both the phenotypic values (arithmetic means of the values obtained from the plants representing a single F_4_ line) obtained from the three environments (Y1, Y2, and Y3) and their average values (AV) were used for QTL mapping analyses. The logarithm of the odds (LOD) threshold of 3.0 was selected for each trait to declare the presence of a significant QTL at *p* < 0.05. The QTL detected in more than two environments were defined as frequent QTL (FQ-QTL). A QTL cluster was defined as two or more traits with significant QTLs having overlapping confidence intervals (*CI*s). Nomenclature for the QTL was denoted as, for instance, *qDFF.pau_4.1* where “qDFF” represents the QTL for days to female flowering, “pau” represents institution name, and 4.1 represents the first QTL on chromosome 4.

## Results

### Phenotypic Variation and Correlation Analysis

The parents of the population, Punjab-14 and PAUBG-6 showed significant differences for most of the investigated traits. PAUBG needs more DFF, DMF, NFFF, and DFM than Punjab-14 which is an early maturing variety. Moreover, Punjab-14 had lower FL, FD, and FW than PAUBG-6. The seeds in PAUBG-6 are harder and more in number than Punjab-14 (Table 1). All nine investigated traits in each environment (including AV) exhibited a continuous distribution (Supplementary Figure 1). The results of the ANOVA showed that the variance of the genotype and the environmental effects of the nine investigated traits were significant. Heritability was calculated using the genotypic and phenotypic coefficient of variation which was high for all the characters (Table 2).

**TABLE 1:** Phenotypic performance of the F_4_ population under Y1, Y2, Y3, and their average values (AV) environments.

Traits	Treatments	Mean Punjab-14	Mean PAUBG-6	Population mean	Skewness	Kurtosis	Range	SD^j^	CV^k^ (%)	Min	Max
DFF^a^	Y1	25.0	40.0	40.5	–0.19	–0.71	28	6.76	16.70	27	55
	Y2	30.0	38.0	35.18	0.82	1.46	9	1.56	4.44	32	41
	Y3	27.0	36.0	38.85	0.12	–0.45	25	5.40	13.90	25	50
	AV	27.3	38.0	38.65	–0.03	–0.12	24	4.83	12.49	26	50
DMF^b^	Y1	25.0	30.0	32.12	0.33	–0.67	33	8.43	26.24	17	50
	Y2	20.0	35.0	34.99	1.14	3.33	11.8	1.82	5.20	29.2	41
	Y3	22.0	33.0	31.56	0.27	–0.15	28	6.23	19.74	18	46
	AV	22.3	32.7	33.45	0.77	0.13	27	6.13	18.32	23	50
NFFF^c^	Y1	12.0	25.0	19.63	0.61	1.2	25	4.37	22.29	9	34
	Y2	15.0	23.0	19.45	0.02	–0.5	31	6.85	35.21	3	34
	Y3	10.0	21.0	18.83	–0.06	0.82	30	4.93	26.20	3	33
	AV	12.3	23.0	19.28	0.18	0.68	25.33	4.54	23.56	8.33	33.67
DFM^d^	Y1	21.0	30.0	23.18	0.13	0.4	10	1.70	7.35	18	28
	Y2	22.0	29.0	22.19	0.62	0.89	11	2.08	9.36	19	30
	Y3	22.0	28.0	22.04	2.2	9.24	18.33	2.51	11.41	17.67	36
	AV	21.7	29.0	22.49	0.51	0.39	9.67	1.74	7.74	18.33	28
FL^e^	Y1	4.0	10.0	105.13	0.17	–0.27	105.8	18.31	17.41	49.96	155.76
	Y2	8.0	15.0	123.52	0.14	0.42	158	15.96	12.90	55	213
	Y3	7.0	13.0	125.72	0.11	0.03	158	20.86	16.59	57	215
	AV	6.3	12.7	119.25	–0.02	0.21	131.11	19.23	16.12	57.02	188.13
FD^f^	Y1	4.5	8.0	39.75	0.37	0.48	32.96	5.71	14.37	26.07	59.02
	Y2	5.0	7.0	44.46	–0.1	0.58	45	8.04	18.07	18	63
	Y3	3.5	8.0	45.82	0.1	–0.52	37.81	8.56	18.69	29	66.81
	AV	4.3	7.7	43.58	0.07	–0.61	28.22	6.52	14.95	30.45	58.67
FW^g^	Y1	30.0	70.0	55.99	0.06	–0.71	73.5	17.77	31.74	21.5	95
	Y2	29.0	80.0	92.75	0.79	0.12	193	25.89	27.90	20	213
	Y3	40.0	77.0	94.18	1.05	0.97	229	29.77	31.60	22	251
	AV	33.0	75.7	81.2	0.69	0.04	148.33	25.55	31.46	22	170.33
SH^h^	Y1	5.0	7.1	5.86	0.41	0	11.28	2.27	38.79	1.4	12.68
	Y2	4.9	10.0	5.89	0.21	–0.39	9.93	2.15	36.55	1.63	11.56
	Y3	3.8	13.0	6.05	0.3	–0.37	10	2.14	35.42	1.9	11.9
	AV	4.6	10.0	6.04	0.3	–0.26	10.65	2.21	36.64	1.64	12.29
SN^i^	Y1	12.0	25.0	14.89	0.68	0.16	28	5.64	37.90	5	33
	Y2	10.0	30.0	15.79	0.51	–0.58	23	5.30	33.55	7	30
	Y3	14.0	27.0	17.26	0.64	0.4	35	6.53	37.82	5	40
	AV	12.0	27.3	15.45	0.43	–0.5	24.67	5.38	34.81	5	29.67

*^a^Days to appearance of first female flower, ^b^Days to appearance of first male flower, ^c^Node to appearance of first female flower, ^d^Days to fruit maturity, ^e^Fruit length (in cm), ^f^Fruit diameter (in cm), ^g^fruit weight (in gm), ^h^seed hardness (in Kgf), and ^i^seed number, ^j^SD, ^k^coefficient of variance.*

**TABLE 2:** Estimates of variance components and heritability for nine traits.

Trait	GV^j^	PV^k^	GEIV^l^	P^m^ value	h^2n^	GCV^o^	PCV^p^
DMF^a^	126.1312	133.3842	33.28266	8.04E-84	0.945623	34.82645	35.81378
DFF^b^	73.89188	80.75601	32.21825	2.95E-79	0.915002	22.51468	23.5372
NFFF^c^	128.9757	135.5395	28.45142	1.35E-83	0.951572	58.90336	60.38362
DFM^d^	15.24979	17.80497	5.86873	1.44E-25	0.856491	17.29923	18.69242
FL^e^	3370.17	3463.439	552.247	8.4E-220	0.97307	49.7002	50.38323
FD^f^	218.3734	228.6489	54.78104	5.5E-117	0.95506	34.25077	35.04734
FW^g^	6328.699	6614.018	1703.971	1.5E-261	0.956862	100.0453	102.2757
SH^h^	24.15951	25.19946	0.862589	0.042754	0.958731	86.84437	88.69379
SN^i^	151.2314	156.4233	26.63008	1.2E-120	0.966809	75.63418	76.9215

*^a^Days to appearance of first female flower, ^b^Days to appearance of first male flower, ^c^Node to appearance of first female flower, ^d^Days to fruit maturity, ^e^Fruit length (in cm), ^f^Fruit diameter (in cm) and ^g^fruit weight (in gm), ^h^seed hardness (in Kgf) and ^i^seed number, ^j^genotypic variance, ^k^phenotypic variance, ^l^genotype × environment interaction variation, ^m^probability, ^n^heritability, ^o^genotypic coefficient of variation, and ^p^phenotypic coefficient of variation.*

**FIGURE 1:**
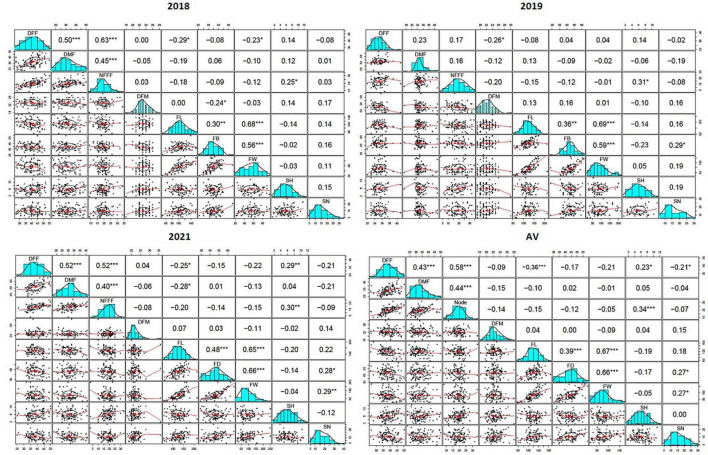
The Pearson’s correlation coefficients (*r*) between nine horticultural traits DFF- days to appearance of first female flower, DMF- days to appearance of first male flower, NFFF- node to appearance of first female flower, DFM- days to fruit maturity, FL- fruit length, FD- fruit diameter and FW- fruit weight, SH- seed hardness and SN- seed number detected in 2018, 2019, and 2021, and average value (AV) environments, *, **, *** indicates level of significance at P ≤ 0.05, 0.01, 0.001 respectively.

A scatterplot displayed the strength, direction, and form of the relationship between two quantitative traits. In this study, it has been observed that there was a moderate, positive, and linear relationship among the earliness related traits (DFF, DMF, and NFFF). The Pearson’s correlation coefficients (*r*) among earliness related traits (DFF, DMF, and NFFF) were significant at 0.05 level of significance (Figure 1). Similarly, most Pearson’s correlation coefficients (*r*) among the fruit related traits (FL, FD, and FW) were significant (Figure 1) and also displayed moderate, positive, linear relationship among the traits. In addition, the *r* values between the earliness and fruit related traits were non-significant.

### Linkage Map and Quantitative Trait Loci Analysis

The information for the SNP markers was obtained from the high-density GBS-based linkage map developed for mapping YMD resistance (Kaur et al., 2021). In that study, 3,144 markers were mapped to the linkage map. The map spanned a total map length of 2,415.2 cM and included 15 linkage groups covering all 11 chromosomes with a mean marker interval of 0.7 cM, mean marker density (marker/cM) of 1.30, and an average of 209.6 markers for each linkage group. Phenotypic data collected for three years for F_4_ population along with respective genotypic data were used for the identification of QTLs for earliness, fruit, and seed related traits. In the present study, a total of 50 QTLs (19 QTLs with LOD > 3.0) for nine traits were detected, which were distributed on 10 out of 11 chromosomes. The QTLs explained 5.09–29.82% of the phenotypic variance. The highest LOD score for a single QTL was 8.68 and the lowest was 2.50. The detail information on the detected QTLs has been given in Table 3.

**TABLE 3:** Summary of the quantitative trait loci (QTLs) detected in the mapping population derived from the cross of bitter gourd cv. Punjab-14 and PAUBG-6 based on SNP based linkage map consisting of 3,144 markers.

Trait	QTL	Chr	LG	Environment	Interval (in cM)	QTL position	LOD^a^	PV^b^	Add^c^	Marker interval	Status
DFF	*qDFF.pau_5.1*	5	6	Y1	143.75–157.31	148.2	3.06	10.00	−2.3	C5_8735936–C5_8770358	
	*qDFF.pau_7.1*	7	9	Y3	86.62–94.72	88.0	4.39	13.20	2.5	C7_15587459–C7_13582712	
	*qDFF.pau_8.1*	8	11	Y1; AV	91.87–104.35	101.0	2.52; 3.39	7.60–11.08	1.3; 2.5	C8_20222740–C8_13998982	*
DMF	*qDMF.pau_5.1*	5	6	Y1; AV	23.6–39.3	34.3	3.57; 2.78	5.95; 5.09	2.2; 1.4	C5_12741217–C5_13902010	*
	*qDMF.pau_7.1*	7	9	Y1; AV	34.23–48.6	38.1	3.54; 3.25	7.23; 6.56	2.3; 1.6	C7_4914632–C7_13838459	*
	*qDMF.pau_7.2*	7	9	Y2	128.6–149.8	136.7	4.35	16.78	0.8	C7_17721806–C7_16438184	
	*qDMF.pau_9.1*	9	12	Y1; AV	53.3–62.27	60.4	3.47; 2.53	8.37; 5.78	2.8; 2.6	C9_20842932–C9_8380053	*
	*qDMF.pau_9.2*	9	12	Y1; Y3; AV	241.1–267.15	241.0	8.68; 6.65; 8.52	17.64; 20.35; 20.27	4.5; 4.0; 3.4	C9_3299961–C9_2982243	*
	*qDMF.pau_9.3*	9	12	Y3	108.66–124.01	112.5	3.53	8.91	−3.0	C9_8527184–C9_7894359	
	*qDMF.pau_11.1*	11	15	AV	361.84–375.41	369.5	2.73	5.50	1.6	C11_10731597–C11_11522926	
NFFF	*qNFFF.pau_3.1*	3	3	Y3; AV	59.0–77.83	67.0	3.31; 4.59	10.08; 13.00	1.7; 1.8	C3_1563894–C3_964192	*
	*qNFFF.pau_9.1*	9	12	Y2	62.27–75.46	68.7	4.11	13.74	2.6	C9_8380053–C9_82738915	
	*qNFFF.pau_10.1*	10	13	Y3; AV	0.0–11.30	9.6	7.3; 4.19	23.41; 12.90	2.4; 2.0	C10_2153284–C10_1011393	*
	*qNFFF.pau_11.1*	11	15	Y1; Y3	197.80–220.56	213.8	4.14; 3.06	12.34; 8.15	1.7; 1.4	C11_6273910–C11_8106029	*
	*qNFFF.pau_11.2*	11	15	Y2	320.41–334.05	330.4	2.61	8.17	2.1	C11_9554901–C11_10337460	
DFM	*qDFM.pau_4.1*	4	4	Y3	35.79–44.50	44.1	3.36	13.10	0.9	C4_4499566–C4_4668198	
	*qDFM.pau_7.1*	7	9	Y1	86.62–105.91	92.9	2.76	9.22	−0.5	C7_15587459–C7_15907575	
	*qDFM.pau_9.1*	9	12	Y1	203.46–220.62	212.2	3.94	12.71	0.6	C9_10957278–C9_21984883	
	*qDFM.pau_11.1*	11	15	Y2	21.81–28.62	26.2	4.03	14.57	−1.0	C11_22738466–C11_18741600	
	*qDFM.pau_11.2*	11	15	Y2; AV	89.34–119.66	115.1	3.10; 3.75	8.48; 12.92	0.6; 0.8	C11_11791232–C11_11279130	*
	*qDFM.pau_11.3*	11	15	Y2	375.41–387.64	385.6	4.78	16.97	1.2	C11_11522926–C11_21543523	
	*qDFM.pau_11.4*	11	15	AV	139.70–154.48	149.5	2.91	10.28	−0.6	C11_11820239–C11_12778880	
FL	*qFL.pau_3.1*	3	3	Y2; Y3; AV	133.98–144.93	140.7	6.68; 4.04; 5.13	20.75; 12.45; 15.78	−13.2; −12.6; 10.6	C3_8939841–C3_11081281	*
	*qFL.pau_8.1*	8	11	Y1	0.0–4.98	0.0	3.77	13.33	9.7	C8_10427890–C8_10489802	
	*qFL.pau_8.2*	8	11	Y2; AV	91.87–104.35	101.0	3.44; 3.26	11.34; 9.46	−10.1; −8.3	C8_20222740–C8_13998982	*
	*qFL.pau_8.3*	8	11	Y3	26.97–44.92	32.2	3.60	10.30	−10.0	C8_13162360–C8_11586888	
	*qFL.pau_10.1*	10	14	Y1	254.7–284.27	267.7	2.50	9.09	−8.1	C10_20471005–C10_21682571	
	*qFL.pau_11.1*	11	15	Y2; Y3	8.30–21.81	11.9	3.84; 2.92	12.38; 15.78	−11.1; −10.6	C11_22044851–C11_22738466	*
FD	*qFD.pau_3.1*	3	3	Y2	82.68–91.07	88.9	2.88	9.42	−3.0	C3_1640344–C3_2945677	
	*qFD.pau_8.1*	8	11	Y2; Y3; AV	26.97–53.95	40.5	7.73; 3.24; 3.70	29.82; 10.28; 13.16	−5.1; −3.4; −2.6	C8_13162360–C8_23546842	*
	*qFD.pau_9.1*	9	12	Y1	263.12–279.01	270.10	2.69	12.17	2.1	C9_3234770–C9_12409897	
	*qFD.pau_9.2*	9	12	Y2	311.71–323.5	315.5	3.42	11.98	−3.0	C9_4484562–C9_4622622	
	*qFD.pau_11.1*	11	15	Y3; AV	89.34–108.45	94.3	3.85; 2.61	13.37; 8.79	−3.2; −2.0	C11_11791232–C11_11554637	*
FW	*qFW.pau_2.1*	2	2	Y2; Y3	8.92–26.67	23.3	5.69; 3.10	20.86; 10.56	−21.6; −16.7	C2_8510111_ C2_8904958	*
	*qFW.pau_6.1*	6	7	Y2	62.09–70.74	69.3	2.92	8.63	13.7	C6_2475888–C6_2611028	
	*qFW.pau_7.1*	7	9	Y1	122.72–136.4	132.6	2.70	10.19	−5.9	C7_17583801–C7_17256691	
	*qFW.pau_8.1*	8	11	Y1	0.0–4.98	1.0	2.52	8.21	6.5	C8_10427890–C8_10489802	
	*qFW.pau_8.2*	8	11	AV	26.97–44.92	40.5	3.94	12.14	−12.6	C8_13162360–C8_11586888	
SH	*qSH.pau_3.1*	3	3	Y1; Y2	0.0–12.79	7.2	2.74; 4.65	8.68; 18.2	2.7; 0.9	C3_973323–C3_816411	*
	*qSH.pau_9.1*	9	12	Y2	26.82–39.5	31.8	3.11	11.35	2.9	C9_6043967_ C9_11311490	
	*qSH.pau_9.2*	9	12	Y3; AV	0.0–8.07	0.0	3.05; 3.08	8.86; 8.98	0.7; 0.7	C9_7113080–C9_6893732	*
	*qSH.pau_9.3*	9	12	Y3; AV	105.45–114.96	111.5	3.97; 4.44	11.38; 12.84	−0.8; −0.8	C9_8527184–C9_8379760	*
	*qSH.pau_10.1*	10	13	Y3; AV	99.31–106.97	104.2	3.60; 3.38	10.46; 9.99	0.7; 0.7	C10_4248989–C10_3363889	*
	*qSH.pau_11.1*	11	15	Y1	115.05–133.33	122.5	2.94	9.52	2.9	C11_10999707–C11_11646977	
SN	*qSN.pau_5.1*	5	6	Y1	82.40–98.16	92.4	2.82	9.72	1.8	C5_19866514–C5_16166935	
	*qSN.pau_7.1*	7	10	Y1; Y2	0.0–11.38	7.9	3.59; 3.33	13.52; 11.97	2.1; 1.8	C7_1604397– C7_15547410	*
	*qSN.pau_7.1*	7	8	Y3	19.06–36.30	28.1	4.01	15.88	−3.1	C7_13874201–C7_10958653	
	*qSN.pau_9.1*	9	12	Y3	180.42–191.94	183.7	2.60	10.22	2.1	C9_10225758– C9_21698372	
	*qSN.pau_9.2*	9	12	AV	31.76–42.89	38.3	3.57	11.90	1.9	C9_6078727_ C9_21460932	
	*qSN.pau_10.1*	10	14	Y2	254.7–284.27	272.4	3.10	12.50	−2.0	C10_20471005– C10_21682571	

^a^Logarithm of odds, ^b^phenotypic variance, ^c^additive effect, and “*’ represents frequent QTLs (FQ-QTLs).

**FIGURE 2:**
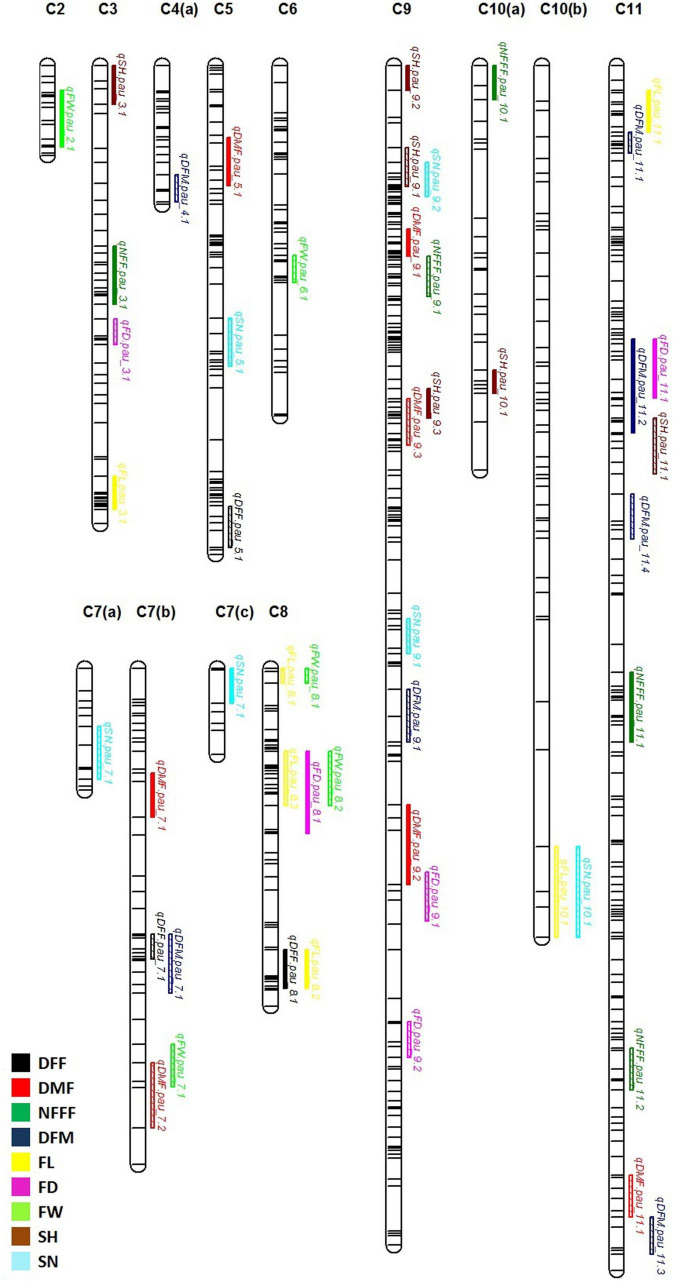
Quantitative trait locus (QTL) detected for nine horticultural traits DFF, DMF, NFFF, DFM, FL, FD and FW, SH and SN detected in environments 2018, 2019, 2021, and AV. Plots indicate the genetic coordinate on the *x*-axis and logarithm of the odds (LOD) score on the *y*-axis of detected QTLs.

For earliness related traits (DFF, DMF, NFFF, and DFM), a total of 22 QTLs were identified on chromosomes 3, 4, 5, 7, 8, 9, 10, and 11 (Figure 2) explaining 5.09–23.41% phenotypic variation (PV). Three QTLs for DFF were identified on chromosomes 5, 7, and 8. On the other hand, seven QTLs for DMF were detected on chromosomes 5, 7, 9, and 11. Similarly, for NFFF five QTLS were detected on chromosomes 3, 9, 10, and 11. Another earliness trait DFM has been mapped on chromosomes 4, 7, 9, and 11. Fruit related traits (FL, FD, and FW) have been mapped on chromosomes 2, 3, 6, 7, 8, 9, and 11. A total of 16 QTLs explaining 8.29–29.82% PV have been identified. For FL, six QTLs have been detected on chromosomes 2, 3, 6, 7, 8, and 9 while for FD, five QTLs have been identified on chromosomes 3, 8, 9, and 11. Four QTLs for FW have been mapped on chromosomes 2, 6, 7, and 8 (Figure 2). Two seed related traits (SH and SN) have been mapped in this study. For these two traits, 12 QTLs were detected on chromosomes 3, 5, 7, 9, 10, and 11 (Figure 2). Six QTLs for SH have been identified on chromosomes 3, 9, 10, and 11. On the other hand, six QTLs for SN have been detected on chromosomes 5, 7, 9, and 11.

### Frequent Quantitative Trait Loci

**FIGURE 3:**
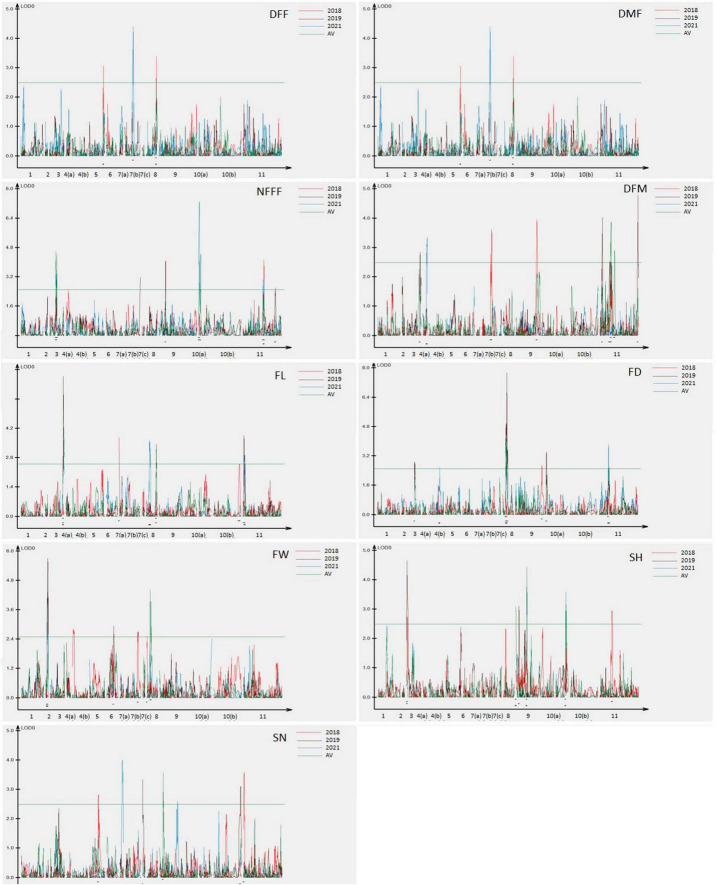
Locations of QTLs for nine horticultural traits based on F_4_ population derived from a cross between Punjab-14 and PAUBG-6. The solid bars represent the QTLs in single environment while bars with lines represents frequent QTLs (FQ-QTLs).

For the earliness related traits, a total of 22 QTLs were detected (Figure 3). Out of these, 12 QTLs were detected only in single environment while 9 QTLs were detected in more than one environment considered as FQ-QTLs. All the frequent QTLs, showed positive additive effects, showing Punjab-14 increasing the effects of the QTL. For the fruit related traits, a total of 16 QTLs were detected (Figure 3), 10 out of 16 QTLs were detected only in single environment while 6 FQ-QTLs were detected. All the frequent QTLs, showed positive additive effects, showing Punjab-14 increasing the effects of the QTL. A total of 12 QTLs were detected for seed related traits (Figure 3) of which 7 QTLs were detected only in single environment while 5 QTLs were FQ-QTLs. Among all the FQ-QTLs identified in the study, 13 showed positive additive effects, with Punjab-14 increasing the effects of the QTL, while seven had negative additive effects, with PAUBG-6 increasing the effects of the QTLs.

### Overlapping Quantitative Trait Loci

Thirteen QTLs regions located on chromosomes 7, 8, 9, 10, and 11 were observed to affect multiple traits in this study (Figure 3). On chromosome 7, two regions containing QTLs for multiple traits were observed. One region contains QTLs for DFM and DFF while other contains QTL for FW and DMF. Similarly, chromosome 8 has three regions controlling multiple traits. Such QTLs included overlapping QTLs for FW, FL, FD, and DFF. Four overlapping QTLs were detected on chromosome 9. These overlapping QTLs control traits, such as SN, SH, NFFF, DMF, and FD. Chromosome 10 has one overlapping QTL for SN and FL traits while chromosome 11 has three overlapping QTLs controlling FL, FD, DFM, DMF, and FD traits.

## Discussion

Inheritance of all fruit traits and yield under study had shown continuous variation and it indicates that the traits were polygenic. The interaction effect between genotypes and environments for all the traits is significant in this study. It has been established that the most important agronomic and horticultural traits in plants, such as yield, early maturation, quality related traits, and plant commercial traits are quantitative traits controlled by both polygenesis and environmental factors. Marker-assisted selection (MAS), based on useful markers and the complex quantitative trait analysis, has been proved to be a suitable and successful breeding method. While genetic and QTL studies have been widely reported for some cucurbit, crops such as cucumber (Wei et al., 2016; Win et al., 2017), bottle gourd (Wu et al., 2017), pumpkin (Zhang et al., 2015), watermelon (Guo et al., 2015; Zhang et al., 2016; Gao et al., 2018), and melon (Chang et al., 2017; Daley et al., 2017; Sheng et al., 2017), such studies for bitter gourd, a non-model plant, were very limited. To date, there have been a few reports on the identification of QTLs for horticulture traits in bitter gourd (Kole et al., 2012; Wang and Xiang, 2013; Cui et al., 2018; Rao et al., 2018, 2021). In the present study, we identified a total of 50 putative QTLs located on 10 chromosomes using the F_4_ population from Punjab-14 and PAUBG-6. The LOD threshold of 3.0 was set, but many QTLs were present in single environments at high LOD value while they were present at low LOD value in other environments so QTLs above LOD value 2.5 were included to observe the frequent occurrence of the QTLs and identification of stable QTLs in different environments. These QTLs were associated with 9 horticulture traits including four earliness related traits, three fruit related traits, and two seed related traits. Each horticulture trait was associated with an average of 5.5 QTLs. Recently, an average of 3 QTLs for six traits (fruit length, fruit diameter, fruit weight, fruit flesh thickness, number of fruits per plant, and yield per plant) in bitter gourd were reported by Rao et al. (2021). In cucurbits, an average of 4–6 QTLs for quantitative traits has been identified in many reports. In *Cucumis sativus*, 36 QTLs for seven traits (cotyledon length, cotyledon width, hypocotyl length, first true leaf length, first true leaf width, aboveground fresh biomass, and aboveground dry biomass at seedling stage) with an average of ∼5 QTL for each trait (Wang et al., 2016) and 19 QTLs for flowering time, fruit size, fruit number, and fruit weight per plant (Sheng et al., 2019) were mapped. Montero-Pau et al. (2017) identified 48 QTLs for vine, flowering, and fruit quality traits in *Cucurbita pepo*. In the present study, three QTLs (for DMF, FL, and FD traits) were detected in three environments with high LOD value, 17 QTLs were identified in two environments. These QTLs can be considered stable while the other 30 QTLs were detected in single environment.

For getting high yield in bitter gourd, early flowering is one of the most important horticultural traits. Early flowering is irregular and affected by genetic, hormonal, and environmental factors (Behera et al., 2010). The traits, such as DFF, DMF, and DFM directly contributed to the earliness of the variety. We identified three QTLs for the DFF trait in which one QTL was detected as FQ-QTL. In a previous study, Rao et al. (2018) mapped five QTLs for the same trait. Seven QTLs had been mapped for DMF in this study. Four QTLs out of seven are considered as FQ-QTL. So far, first male flower appearance locus has not been mapped in bitter gourd. For production of gynoecious hybrid cultivars of bitter gourd, monoecious lines with early male flowering have high potential as pollenizers (Dhillon et al., 2017). So, the information in this study will be helpful in future to develop varieties with early male flowering.

Nodes at which the female flower appear clearly indicate both maturity and sex tendency of the plant. For example, lower the number of node for the appearance of first female flower, earlier the bearing of fruit (Shiefriss and Galun, 1956; Dey et al., 2006). As reported by Trivedi (1983), lower node of first female flower appearance indicates the earliness of the variety while higher the node greater the production of female flower and shorter the interval between male and female flowering resulting in short plant life. This information can help in improving the cropping method, fruit quality, and breeding strategies (Miniraj et al., 1993). In this study, a total of five QTLs were detected for NFFF in which three were observed as frequent QTLs. In previous studies, Wang and Xiang (2013) identified three QTLs, Cui et al. (2018) mapped two QTLs, and Rao et al. (2018) identified two QTLs for the same trait. The exact timing of harvest of bitter gourd fruit is difficult to determine as its fruits are consumed in unripe condition (before fruits attain maturity). The ripe fruits are unmarketable. Behera et al. (2010) reported that when fruits slightly change their color and exocarp (ridges and bumps) develops, indicates the optimal time of harvest and this condition is hard to ascertain. So, days to fruit maturity data were taken from the date of pollination to the day when fruits turn yellow or yellowish orange. Days taken by fruits to mature hold a major role in earliness of the variety. Lesser the days taken by fruits to mature means earlier yield can be expected. In this study, seven QTLs were detected for DFM with only one FQ-QTL. This is the first report of QTL mapping for days to fruit maturity in bitter gourd.

Fruit size is the direct indicator of yield which is depicted from fruit length, fruit weight, and fruit diameter. Therefore, major priority is given on selection of genotypes having a high average fruit weight, fruit length, and fruit diameter which would lead to the development of high yielding cultivars of bitter gourd (Behera et al., 2010). The six QTLs for fruit length have been detected in this study. Three QTLs were the FQ-QTL identified for the trait in this study. Five QTLs for fruit diameter was mapped on chromosomes 3, 8, 9, and 11. Five QTLs for fruit weight were present on chromosomes 2, 6, 8, and 7 (Figure 2). In 2012, Kole et al. (2012) mapped QTLs for fruit traits and found two QTLs on LG7 and LG2 for fruit length. Wang and Xiang (2013) also mapped fruit traits and detected four QTLs for fruit weight.

Seed hardness is an important trait in bitter gourd. The seed emergence in field is always problematic in bitter gourd due to hard seed coat. The seeds with thick seed coat do not absorb water properly which results in delay in germination. That is why; it is advised to plant seedlings because seed coat dormancy creates problem and seed germination rate is not 100% which leads to the loss of seeds (Pandita and Nagarajan, 2004). In addition, in some cases, pre-sowing treatment (priming) is given by mixing the seeds with moistened vermiculite and storing at 20°C for 36 h or keeping the seeds in hot water at 40°C for 4 h have been suggested, for the successful germination under suboptimal temperature (Lin and Sung, 2001; Hsu et al., 2003). Six loci responsible for seed hardness in bitter gourd had been mapped on chromosomes 3, 9, 10, and 11. This is the first report on the mapping of seed hardness in bitter gourd so far. Seed is the start and end points of plant life, and the important determinants of growth and development. Seediness (number of seeds per plant) helps the plant in producing a greater numbers of offspring to survive. Varieties with high fruit seed weight and fruit seed number are preferred not only to increase the crop production, but also to meet the needs of the seed industry and farmers. As compared with other cucurbit crops, bitter gourd produces a smaller number of seeds. Six QTLs for seed number in bitter gourd had been mapped in the present study (Figure 2).

The colocalization of QTLs for different traits was frequently reported in previous studies (Groos et al., 2003; Li et al., 2007). In this study, several QTLs detected were co-localized and some were clustered (Figure 3). QTLs controlling flowering-related traits, fruit related traits, and seed related traits were found overlapping at many locations on chromosome 7, 8, 9, 10, and 11. Such linkage could be substantiated by their correlation coefficient value (Figure 1). Similarly, high correlation among the fruit diameter, fruit length, and weight was reflected in the similarity of their genomic location of QTLs controlling each of them. Understanding phenotypic components that are associated with the fruit yield will be important toward breeding for yield. A study in melon (Zalapa et al., 2007) confirms that fruit number and yield do share overlapping genomic regions underlying the traits. In support to the overlapping QTLs between fruit weight and diameter in our study, Yuan et al. (2008) showed that fruit weight, length, and diameter do have overlapping QTLs. In previous studies in bitter gourd, the colocalization of QTLs has been well documented (Kole et al., 2012; Wang and Xiang, 2013). Yuan et al. (2008) showed overlapping QTLs between fruit weight and diameter in cucumber. In these studies, the QTLs for fruit traits which contribute toward the yield has been found in overlapping and clusters.

## Conclusion

In this study, we used genotyping by sequencing SNP-based linkage map to identify QTLs associated with earliness, fruit, and seed traits in bitter gourd. A total of 50 QTLs for nine horticultural traits were detected based on the genetic linkage map. Of these, 30 QTLs were detected in only one environment while 20 were detected in more than two environments. The information generated in this study will provide a basis for MAS and map-based cloning in further studies.

## Data Availability Statement

The datasets presented in this study can be found in online repositories. The names of the repository/repositories and accession number(s) can be found below: https://www.ncbi.nlm.nih.gov/, PRJNA702876.

## Author Contributions

GK and GC performed phenotyping. DS contributed in SNP identification. NK and MP designed and supervised the project. GK and NK wrote and edited the manuscript. PC gave critical suggestions throughout the study and edited the manuscript. All authors contributed to the article and approved the submitted version.

## Conflict of Interest

The authors declare that the research was conducted in the absence of any commercial or financial relationships that could be construed as a potential conflict of interest.

## Publisher’s Note

All claims expressed in this article are solely those of the authors and do not necessarily represent those of their affiliated organizations, or those of the publisher, the editors and the reviewers. Any product that may be evaluated in this article, or claim that may be made by its manufacturer, is not guaranteed or endorsed by the publisher.

## Footnotes

1
https://www.r-project.org/
