# Supplementary material for: Quantitative Trait Loci Mapping for Earliness, Fruit, and Seed Related Traits Using High Density Genotyping-by-Sequencing-Based Genetic Map in Bitter Gourd (Momordica charantia L.)
Source: Front Plant Sci. 2022 Feb 8;12:799932. doi: 10.3389/fpls.2021.799932 (PMC8863046; doi:10.3389/fpls.2021.799932)
Supplement: Supplementary file 1 [file Data_Sheet_1.docx]

***Supplementary Material***

**Supplementary Figures**


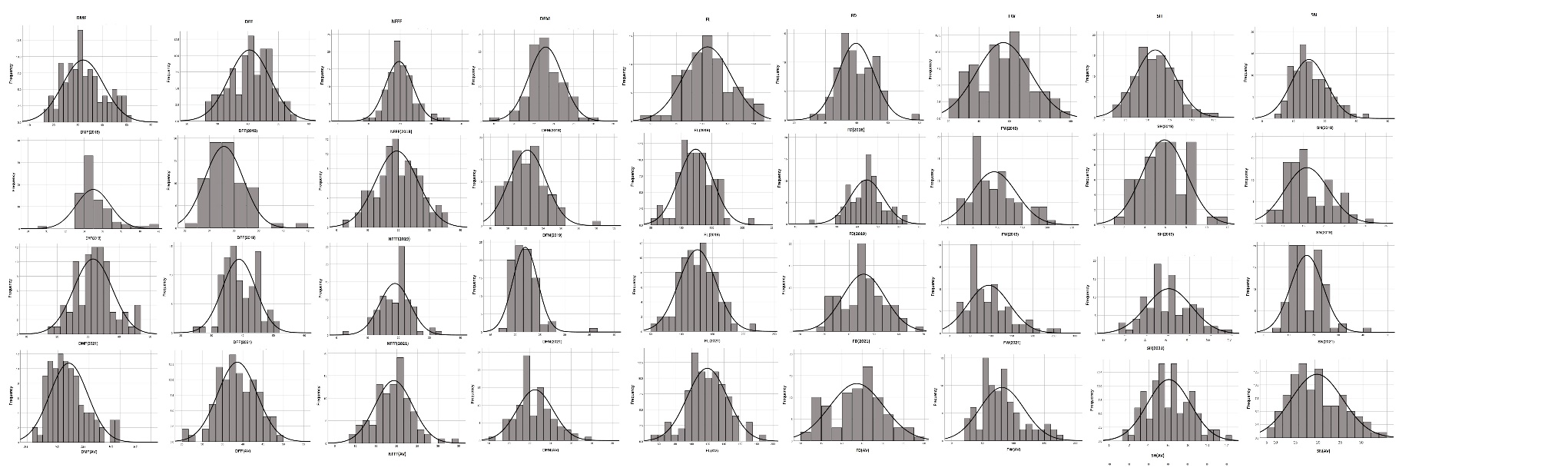


**Supplementary Figure 1.** Frequency distribution for nine horticultural traits (days to appearance of first female flower (DFF), days to appearance of first male flower (DMF), node to appearance of first female flower (NFFF) days to fruit maturity (DFM), fruit length (FL), fruit diameter (FD) and fruit weight (FW), seed hardness (SH) and seed number (SN).) in F_4:5_ families derived from the cross between Punjab-14 and PAUBG-6 in three environments
